# A neural network approach to chemical and gene/protein entity recognition in patents

**DOI:** 10.1186/s13321-018-0318-3

**Published:** 2018-12-18

**Authors:** Ling Luo, Zhihao Yang, Pei Yang, Yin Zhang, Lei Wang, Jian Wang, Hongfei Lin

**Affiliations:** 1 College of Computer Science and Technology, Dalian University of Technology, Dalian, China; 2 Beijing Institute of Health Administration and Medical Information, Beijing, China

**Keywords:** Patents, Biomedical entity recognition, Deep learning, Long short-term memory, Conditional random field

## Abstract

In biomedical research, patents contain the significant amount of information, and biomedical text mining has received much attention in patents recently. To accelerate the development of biomedical text mining for patents, the BioCreative V.5 challenge organized three tracks, i.e., chemical entity mention recognition (CEMP), gene and protein related object recognition (GPRO) and technical interoperability and performance of annotation servers, to focus on biomedical entity recognition in patents. This paper describes our neural network approach for the CEMP and GPRO tracks. In the approach, a bidirectional long short-term memory with a conditional random field layer is employed to recognize biomedical entities from patents. To improve the performance, we explored the effect of additional features (i.e., part of speech, chunking and named entity recognition features generated by the GENIA tagger) for the neural network model. In the official results, our best runs achieve the highest performances (a precision of 88.32%, a recall of 92.62%, and an F-score of 90.42% in the CEMP track; a precision of 76.65%, a recall of 81.91%, and an F-score of 79.19% in the GPRO track) among all participating teams in both tracks.

## Introduction

Biomedical named entity recognition (NER) aims to automatically find the biomedical mentions in text, which is crucial for the information extraction in biomedical domain. In the previous BioCreative challenges [1–3], various tasks have been addressed to recognize biomedical entities (such as gene/protein, chemical and disease) from the scientific literature. In addition to the scientific literature, patents are another important source since they contain a wealth of useful biomedical information. Therefore, automatic extraction of information contained in patents has received much attention, and automatic biomedical entity recognition from medicinal chemistry patents has become an important research task [4].

To promote the development of NER systems, the BioCreative V.5, a major challenge event in biomedical natural language processing, organized three tracks to focus on biomedical entity recognition in patents. This challenge included three individual tracks: two traditional BioCreative tracks to detect relevant biomedical entities (chemical entity mention recognition (CEMP) track and gene and protein related object recognition (GPRO) track) and a novel track called technical interoperability and performance of annotation servers (TIPS). The latter focuses on the technical aspects of the evaluation of continuous text Annotation Servers for NER. For the challenge, we participated in the CEMP and GPRO tracks, and our submissions to the two tracks were created by our deep learning system.

The biomedical NER is a fundamental step for further biomedical text mining and has received much more attention recently. However, biomedical NER is particularly challenging due to some reasons. For example, for gene/protein NER, millions of gene/protein names are used, new names are created constantly and rapidly, gene/protein names naturally co-occur with other types that have similar morphology and context, various ways of naming gene and ambiguities caused by DNA sequences may vary in nonspecific ways [1]. In the previous works, the state-of-the-art CRF-based biomedical NER methods [5–9] depend on effective feature engineering, i.e., the design of effective features using various natural language processing (NLP) tools and knowledge resources, which is still a labor-intensive and skill-dependent task. Recently, deep learning has become prevalent in the machine learning research community. These are neural network-based representation learning methods that compose simple but non-linear modules to obtain multiple levels of representation [10]. For the NER task in general domain (such as news domain), several similar neural network architectures [11–13] have been proposed and exhibit promising results. Moreover, deep learning methods have begun to be explored in biomedical field, including genes and proteins [14], diseases [15] and chemicals [16]. Compared with the traditional machine learning methods, the key advantage of deep learning methods is that these layers of features are not designed by human engineers and, therefore, less feature engineering is needed.

In this paper, we describe our NER systems based on the neural network for the CEMP and GPRO tracks. In the approach, first the word embedding is learned from a large unlabeled dataset. Thereafter, character feature is produced with the character and capitalization embeddings. Then the concatenation of the character feature and the word embedding is used as a basic input. Finally, the input is fed into a bidirectional long short-term memory with a conditional random field layer (BiLSTM-CRF) to recognize chemical and gene/protein entities from patents. Furthermore, we explored the effect of additional features (i.e., part of speech (POS), chunking and NER features generated by the GENIA tagger) for the neural network model. In the official results, our best runs achieve the highest performances (the F-scores of 90.42% and 79.19% on the CEMP and GPRO corpora, respectively) in both tracks. The details of our method and results are presented in the following sections.

## Methods

**Fig. 1:**
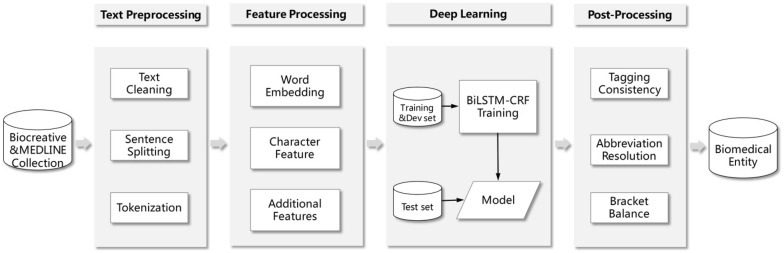
The processing flowchart of our system

Similar to many NER tasks, we modeled the biomedical NER as a sequence labeling problem. We used the BIO (Begin, Inside, Outside) tagging scheme since it achieves better performance than BIOES tagging scheme in our experiments. For the challenge, we present the system based on the neural network architecture (i.e., BiLSTM-CRF) to recognize biomedical entities from patents. The processing flow of our system is shown in Fig. 1. Firstly, some preprocessing steps including text cleaning, sentence splitting and tokenization are performed. Secondly, a word embedding is learned with large amounts of unlabeled data with the word2vec tool. Moreover, we induce the character feature and additional features (such as POS, chunking and NER features generated by the GENIA tagger). Then with the features as input, a BiLSTM-CRF model is trained by the annotated training set. Finally, some post-processing steps including tagging consistency, abbreviation resolution and bracket balance are employed. The process is described in details in the following sections.

### Text preprocessing

First, document titles and abstracts are extracted from the dataset. The extracted text is then split into the sentences, tokenized using the Stanford CoreNLP tool [17]. Note that the tokenization of the Stanford CoreNLP tool does not split text into segments at the dash (-) character. However, in the biomedical documents, some chemical and gene/protein entity names and other words are always combined into one token using dash character. For example, “ephrinB-EphB” is annotated as two entities (i.e., “ephrinB” and “EphB”); “CD3-binders” is only annotated with “CD3” as an entity. To address the cases, we broke the text into separated segments at the dash character (e.g., “ephrinB-EphB” is split into three tokens: “ephrinB”, “-” and “EphB”). The experimental results show that the processing can improve the performance of our system.

### Features

Distributed word embedding and character feature are widely used in the field of NLP, especially in the deep learning methods. We also used them as a basic feature of our NER system. Moreover, to investigate the effects of traditional features (such as POS, chunking, and NER features), these features are added into the model as additional features. All feature embeddings are parameters of the model, and they can be optimized when the model is trained. Table 1 shows an example of all features from tokens corresponding to a sentence. Details of each of features are presented as follows.

**Table 1:** An example of all features

Input	Substituted	piperidines	with	selective	binding	to	histamine	h3	–	receptor	.
Word	substituted	piperidines	with	selective	binding	to	histamine	h3	–	receptor	.
Character	s u b s t i t u d e d	p i p e r i d i n e s	w i t h	s e l e c t i v e	b i n d i n g	t o	h i s t a m i n e	h 3	–	r e c e p t o r	.
Cap	firstCaps	lower	lower	lower	lower	lower	lower	lower	lower	lower	lower
POS	VBN	NNS	IN	JJ	NN	TO	NN	NN	HYPH	NN	.
Chunk	B-NP	I-NP	B-PP	B-NP	I-NP	B-PP	B-NP	I-NP	B-NP	I-NP	O
NER	O	O	O	O	O	O	B-protein	I-protein	I-protein	I-protein	O

#### Word embedding

Word embedding, also known as distributed word representation, can capture both the semantic and syntactic information of words from a large unlabeled corpus and has attracted considerable attention from many researchers [18]. Compared with the bag-of-words representation, word embedding is low-dimensional and dense. In recent years, several models, such as word2vec [19] and GloVe [20], have been proposed and widely used in the field of NLP. To achieve a high-quality word embedding, we downloaded a total of 1,918,662 MEDLINE abstracts from the PubMed website as the unlabeled data. Then the data and all datasets (The training set comprises a total of 21,000 abstracts, and the test set comprises a total of 9000 abstracts.) provided in the BioCreative V.5 CEMP and GPRO tracks were used to train the word embedding by the word2vec tool using the skip-gram model as pre-trained word embedding.

#### Character feature

In addition to the word embedding, character-level features in a name contain rich structure information of the entity. These features (such as character n-grams, prefixed and suffixes) are commonly employed in the current NER methods [21]. Unlike the previous traditional methods in which character features are based on hand-engineering, character embedding can be learned while training. Character embedding has been found useful for many NLP tasks. They can not only learn interior representations of the entity names, but also alleviate the out-of-vocabulary problem [22]. In our model, a bidirectional long short-term memory (BiLSTM) is used to obtain the character-level feature. First, a character lookup table which contains a character embedding for every character is initialized randomly. The sequence of characters in a word is transformed to a sequence of embeddings with fixed length *L*, where *L* is the max length of all words. If the word has a length less than *L*, we pad it with zero embeddings. Then the character embedding corresponding to every character in a word is given in both direct and reverse orders to a BiLSTM. Further, we used a separate lookup table to add a capitalization feature since capitalization information is erased during the word and character embeddings. The capitalization feature is obtained with the following options: allCaps (all characters are uppercase in a word), firstCaps (only the first character is uppercase), lower (all characters are lowercase), others (the other case excluding the above ones). At last, the concatenation of the forward and backward representations from the BiLSTM and the capitalization feature is used as the character-level feature of the word.

#### Additional features

Due to the complexity of the natural language and the specialty of the biomedical domain, some linguistic and domain features are often employed in traditional machine learning methods for biomedical NER [7, 9]. We also explored the effect of linguistic features (such as POS and chunking features). The POS information and chunking information of each word were generated by the GENIA tagger (http://www.nactem.ac.uk/GENIA/tagger/). In addition, named entity tags information (including protein, DNA, RNA, cell line and cell type entities) generated by the GENIA tagger was also used as a feature. And the NER feature of each token was encoded in the BIO tagging scheme. In our experiments, three different lookup tables were to output POS, chunking, and NER embeddings, respectively. And they were initialized randomly.

### BiLSTM-CRF model

**Fig. 2:**
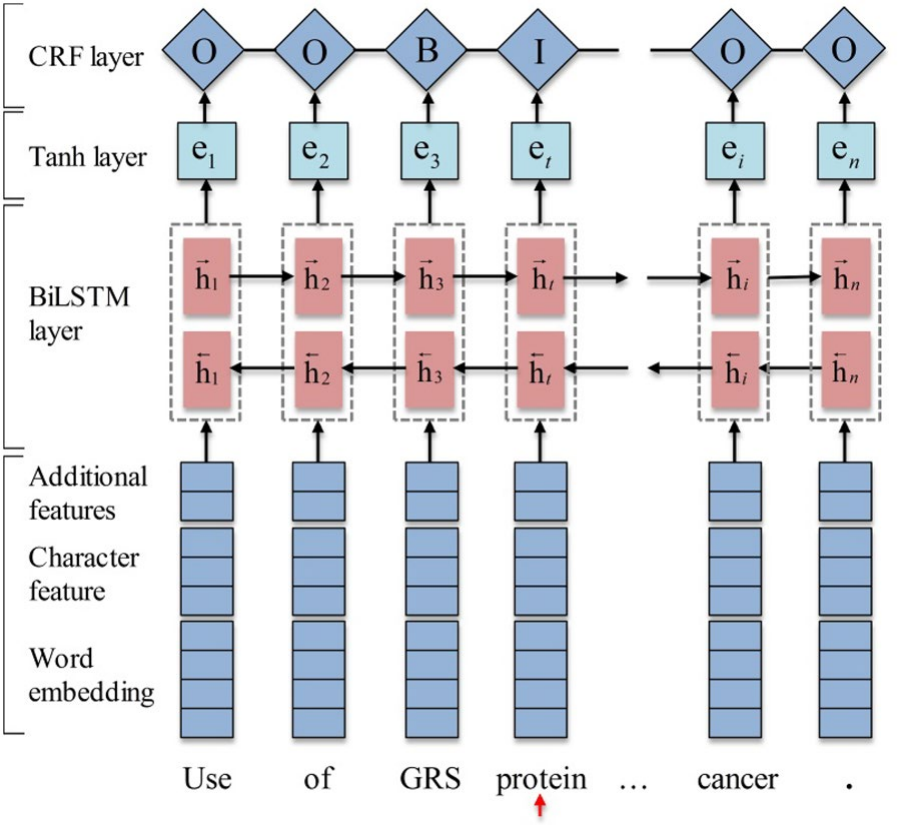
The architecture of BiLSTM-CRF model

Our system is a deep learning one based on a bidirectional long short-term memory model with a conditional random field layer, whose architecture is illustrated in Fig. 2.

Recurrent neural networks (RNNs) are a family of neural networks for processing sequential data. Giving a sequence of vectors ${\mathbf{X}} = ({\mathbf{x}}_{1} ,{\mathbf{x}}_{2} , \ldots ,{\mathbf{x}}_{t} , \ldots ,{\mathbf{x}}_{n} )$ as input, they return another corresponding sequence $({\mathbf{h}}_{1} ,{\mathbf{h}}_{2} , \ldots ,{\mathbf{h}}_{t} , \ldots ,{\mathbf{h}}_{n} )$, where *n* is the length of the sequence. The current state ${\mathbf{h}}_{t}$ is generated from the input ${\mathbf{x}}_{t}$ and the state ${\mathbf{h}}_{t - 1}$ that is passed forward though time. However, traditional RNNs have the mathematical challenge of learning long-term dependencies. The main problem is that gradients propagated over many stages tend to vanish. When the sequence is long, learning long-term dependencies is difficult for traditional RNNs [23]. To alleviate this problem, long short-term memory (LSTM) [24] is designed by incorporating a memory cell with the gating mechanism and has been shown to capture long-range dependencies. Therefore, LSTM is applied in our system. LSTM memory cell is implemented as the following:

1 $${\mathbf{i}}_{t} = \sigma \left( {{\mathbf{W}}^{\left( i \right)} {\mathbf{x}}_{t} + {\mathbf{U}}^{\left( i \right)} {\mathbf{h}}_{t - 1} + {\mathbf{V}}^{\left( i \right)} {\mathbf{c}}_{t - 1} + {\mathbf{b}}^{\left( i \right)} } \right)$$

2 $${\mathbf{c}}_{t} = \left( {1 - {\mathbf{i}}_{t} } \right)*{\mathbf{c}}_{t - 1} + {\mathbf{i}}_{t} *{ \tanh }\left( {{\mathbf{W}}^{\left( c \right)} {\mathbf{x}}_{t} + {\mathbf{U}}^{\left( c \right)} {\mathbf{h}}_{t - 1} + {\mathbf{b}}^{\left( c \right)} } \right)$$

3 $${\mathbf{o}}_{t} = \sigma \left( {{\mathbf{W}}^{\left( o \right)} {\mathbf{x}}_{t} + {\mathbf{U}}^{\left( o \right)} {\mathbf{h}}_{t - 1} + {\mathbf{V}}^{\left( o \right)} {\mathbf{c}}_{t} + {\mathbf{b}}^{\left( o \right)} } \right)$$

4 $${\mathbf{h}}_{t} = {\mathbf{o}}_{t} *{ \tanh }\left( {{\mathbf{c}}_{t} } \right)$$

where $\sigma$ is the element-wise sigmoid function, and $*$ is the element-wise product. $\{ {\mathbf{W}}^{\left( . \right)} ,{\mathbf{U}}^{\left( . \right)} ,{\mathbf{V}}^{\left( . \right)} \}$ is the weight matrix set. $\{ {\mathbf{b}}^{\left( . \right)} \}$ is the bias vector set.

However, the LSTM’s hidden state $${\mathbf{h}}_{t}$$ only takes the information from the left context of the sequence at every time *t*. To learn left and right context information simultaneously, an elegant solution is a bidirectional LSTM (BiLSTM) [25]. In the BiLSTM architecture, a forward LSTM computes a representation $${\vec{\mathbf{h}}}_{t}$$ of the sequence from left to right, and another backward LSTM computes a representation $${\mathbf{\overset{\lower0.5em\hbox{$\smash{\scriptscriptstyle\leftarrow}$}}{h} }}_{t}$$ of the same sequence in reverse. These two distinct networks use different parameters, and then the representation of a word is obtained by concatenating its left and right context representations, i.e., $${\mathbf{h}}_{t} = \left[ {{\vec{\mathbf{h}}}_{t} ;{\mathbf{\overset{\lower0.5em\hbox{$\smash{\scriptscriptstyle\leftarrow}$}}{h} }}_{t} } \right]$$. The representation can make use of rich context information. Then a tanh layer on top of the BiLSTM is used to predict confidence scores for the word having each of the possible labels as the output scores of the network.

5 $${\mathbf{e}}_{t} = \tanh \left( {{\mathbf{W}}^{(e)} {\mathbf{h}}_{t} + {\mathbf{b}}^{(e)} } \right)$$

where the weight matrix ${\mathbf{W}}^{\left( e \right)}$ and the bias vector ${\mathbf{b}}^{\left( e \right)}$ are the parameters of the model to be learned in training.

Similar to many NER tasks, we modeled the biomedical NER as a sequence labeling problem. In the sequence labeling problem, the output labels have strong dependencies. In addition to information of the word itself and the context, the entity tag of the word is also decided by the context tags information of the word. For example, in a reasonable entity tag sequence, the tag “**I**” generally appears after the tag “**B**”, but it does not appear after the tag “**O**”. However, the above-mentioned output scores of the network only use the ${\mathbf{e}}_{t}$ to make independent tagging decisions for each output. Therefore, instead of modelling tagging decisions independently, the CRF layer is added after the tanh layer to decode the best tag path in all possible tag paths. To be more specific, we consider ${\mathbf{P}}$ to be the matrix of scores output by the network. The *t*th column of the matrix is the vector ${\mathbf{e}}_{t}$ obtained by the Eq. (5). The element $P_{i,j}$ of the matrix is the score of the *j*th tag of the *i*th word in the sentence. Moreover, we introduce a tagging transition matrix ${\mathbf{T}}$, where $T_{i,j}$ represents the score of transition from tag *i* to tag *j* in successive words and $T_{0,j}$ as the initial score for starting from tag *j*. This transition matrix will be trained as the parameter of model. The score of the sentence ${\mathbf{X}}$ along with a sequence of predictions ${\mathbf{y}} = ({\text{y}}_{1} ,{\text{y}}_{2} , \ldots ,y_{t} , \ldots ,y_{n} )$ is then given by the sum of transition scores and network scores:

6 $$s\left( {{\mathbf{X}},{\mathbf{y}}} \right) = \mathop \sum \limits_{i = 1}^{n} \left( {T_{{y_{i - 1} ,y_{i} }} + P_{{i,y_{i} }} } \right)$$

Then we use a softmax function to yield the conditional probability of the path ${\mathbf{y}}$ by normalizing the above score over all possible tag paths ${\tilde{\mathbf{y}}}$:

7 $$p\left( {{\mathbf{y}} |{\mathbf{X}}} \right) = \frac{{e^{{s\left( {{\mathbf{X}},{\mathbf{y}}} \right)}} }}{{\mathop \sum \nolimits_{{{\tilde{\mathbf{y}}}}} e^{{s\left( {{\mathbf{X}},{\tilde{\mathbf{y}}}} \right)}} }}$$

During the training phase, the objective of the model is to maximize the log-probability of the correct tag sequence:

8 $$\log p\left( {{\mathbf{y}} |{\mathbf{X}}} \right) = s\left( {{\mathbf{X}},{\mathbf{y}}} \right) - \log \mathop \sum \limits_{{{\tilde{\mathbf{y}}}}} e^{{s\left( {{\mathbf{X}},{\tilde{\mathbf{y}}}} \right)}}$$

At inference time, we predict the best tag path that obtains the maximum score given by:

9 $${ \arg }\mathop {\hbox{max} }\limits_{{{\tilde{\mathbf{y}}}}} s\left( {{\mathbf{X}},{\tilde{\mathbf{y}}}} \right)$$

This can be computed using dynamic programming, and the Viterbi algorithm [26] is chosen for this inference.

### Training procedure

The word embedding of our model is initialized with pre-trained word embedding and other parameters are initialized at random from a uniform distribution. Then all parameters are optimized using stochastic gradient descent (SGD) [27] to maximize the log-probability of the correct tag sequence. In addition, several hyper-parameters need to be determined in our model. We tuned the hyper-parameters on the development set by random search [28]. The main hyper-parameters of our models are shown in Table 2. The number of epochs is chosen by early stopping strategy [29] on the development set. Our model is implemented using open-source deep learning library Theano (http://deeplearning.net/software/theano/) and trained on a NVIDIA Tesla K40 GPU.

**Table 2:** The main hyper-parameters of our model

Hyper-parameter	Value	Values tested
Word embedding dimension	100	50, 100, 200
Character embedding dimension	25	25, 50
Character-level BiLSTM state size	25	25, 50
Capitalization embedding dimension	5	5, 10
POS embedding dimension	25	25, 50
Chunking embedding dimension	10	10, 20
NER embedding dimension	5	5, 10
Word-level BiLSTM state size	100	50, 100, 200
SGD learning rate	0.001	0.01, 0.005, 0.001

### Post-processing

For performance optimization, we also employed several common post-processing steps including tagging consistency, abbreviation resolution and bracket balance.

If the number of a word sequence tagged by our model as a biomedical entity exceeds 50% of the total number of the sequence in a document (title and abstract), all instances of the word sequence will be tagged as an entity mention. For example, if our BiLSTM-CRF model found three gene/protein mentions of “nociception receptor” and missed out two other mentions of “nociception receptor” in a document, the missed mentions would be retrieved.

For abbreviation resolution, all local abbreviation definitions, such as “protease-activated receptor 1 (PAR1)”, will be found. If the abbreviation (i.e., “PAR1”) in the long form was tagged by our model, then all instances of the abbreviation in the document would be tagged.

While there are some mentions with unbalanced brackets (such as parenthesis, square brackets and curly brackets), we attempted to balance the brackets by adding or removing characters to the right or left of the mention. For example, if “OGP(10” (the next characters in the text are “− 14)”) was tagged as an mention by our model, then the mention would be extended to include the right parenthesis (i.e., “OGP(10–14)”). If the unbalanced bracket is the first or last character of the entity tagged by the model (e.g., “(nNOS”), the bracket would be simply discarded.

## Results and discussion

In this section, first the experimental datasets and settings are introduced, and then the experimental results and discussion are presented.

### Experimental datasets and settings

The organizers of the BioCreative V.5 challenge provided the corpora (i.e., the CEMP and GPRO corpora) including the training and test sets. The training set comprises a total of 21,000 manually annotated documents (title and abstract), and test set comprises a total of 9000 unannotated documents. Furthermore, annotations for the GPRO track are divided in two groups: type 1, covering GPRO mentions that can be normalized to a database record; and type 2, covering those GPRO mentions that in principle cannot be normalized to a unique bio-entity database record [30]. Table 3 describes the statistic of the CEMP and GPRO corpora. In our experiments, for the GPRO task, we only consider entities that can be mapped to an identifier (type 1) are evaluated like the GPRO subtask in the BioCreative V does [4], and the type 2 entities are ignored. We randomly selected the 10% of the training set as the development set (Dev) to tune the hyper-parameters and the remaining documents were used to train our system. Only the annotations of the training sets were made available to the participants in the challenge. To evaluate the performance of their system on the test set (Test), teams could submit up to five runs to the BeCalm Web metaserver platform [31]. The micro-averaged recall, precision and F-score statistics were used for final prediction scoring, and F-score was selected as main evaluation metric. The gold-standard annotation of the test dataset has not yet been released by the organizers.

**Table 3:** CEMP and GPRO corpora overview

	Training set	Test set	Entire corpus
Patent abstracts	21,000	9000	30,000
CEMP mentions	99,632	44,486	144,188
GPRO mentions	17,751	8998	26,749
GPRO type 1 mentions	12,422	5330	17,752
GPRO type 2 mentions	5329	3668	8997
Tokens	1,770,836	767,599	2,538,435

### The effect of the different ratios of positive and negative documents

In the CEMP corpus, 16,539 documents in the training set contain annotated chemical entities and the rest 4461 documents do not contain them. However, in the GPRO corpus, only 5795 documents in the training set contain annotated gene/protein entities and the rest 15,205 documents do not contain them. In our experiments, to explore the effectiveness of the documents without annotated biomedical entities, the corresponding corpus was divided into the different training sets by the ratio of positive documents (the documents with annotated biomedical entities) and negative documents (the documents without annotated biomedical entities). First, the negative documents are randomly selected by the ratio. Then they and all positive documents are combined into a new training set. In the experiments, word embedding and character feature are used as the inputs of the BiLSTM-CRF model. The results of the models trained with the different training sets on our development sets are shown in Table 4.

**Table 4:** The effect of the different ratios of positive and negative documents

Ratio (positive:negative)	CEMP Dev			GPRO Dev		
	Precision	Recall	F-score	Precision	Recall	F-score
1:0	87.58	92.20	89.83	60.90	88.27	72.07
1:0.5	–	–	–	66.06	85.76	74.63
1:1	–	–	–	67.97	86.06	*75.95*
1:2	–	–	–	70.03	77.79	73.71
All training set	87.58	92.50	*89.97*	68.32	82.44	74.72

On the CEMP corpus, only the ratio (1:0) and all training set were tested since the number of positive documents is more than the number of negative documents

Italic values denote the highest values

On the CEMP corpus, there is slight difference among the F-scores of these models. The reason is that only small amounts of documents do not contain chemical entities. On the GPRO corpus, the model achieves the best performance with an F-score of 75.95% when the number of positive and negative documents in the training set is equal. When the number of positive documents exceeds the number of negative documents, the more token sequences are predicted as the entities. In this case, the model performs worse owing to a significant drop in precision. When the number of negative documents exceeds the number of positive documents, the model also performs worse owing to a significant drop in recall. In the following experiments, all CEMP training set is used to train the models, while the balanced version of GPRO training set is used.

### The effect of the model components on the development set

In our experiments, the BiLSTM-CRF with the basic feature (i.e., word embedding and character feature) is used as our baseline. To further analyze the effectiveness of our baseline model components, the corresponding experiments are conducted by removing one component each time. Table 5 reports the evaluation results on our development sets.

**Table 5:** The effect of our baseline components on our development sets

Model	CEMP Dev				GPRO Dev			
	Precision	Recall	F-score	△	Precision	Recall	F-score	△
Baseline	87.58	92.50	*89.97*	–	67.97	86.06	*75.95*	–
− Character embedding	86.27	90.98	88.56	− 1.41	66.67	83.69	74.22	− 1.73
− Capitalization feature	87.99	91.42	89.67	− 0.30	68.07	84.94	75.58	− 0.37
− CRF layer	84.84	88.55	86.66	− 3.31	62.81	79.41	70.14	− 5.81
− Post-processing	87.30	92.28	89.72	− 0.25	68.04	85.61	75.82	− 0.13

Italic values denote the highest values

The similar results were observed on both CEMP and GPRO corpora. The results show that each component makes different degrees of contribution. Among others, the CRF layer makes the most significant contribution. After the CRF layer is removed, the F-score decreases by 3.31% and 5.81% on the CEMP and GPRO development sets, respectively. It demonstrates that BiLSTM has the ability of handing sequential data and learning the long-range context information, but the performance of the model can still be further improved by considering the dependencies of output labels (which is implemented with the CRF layer). In addition, the character embedding is also important. Removing the character embedding leads to the decrease of F-score by 1.41% and 1.73% on the CEMP and GPRO development sets, respectively. The reason is that character information can not only capture interior representations of the entity names, but also alleviate the out-of-vocabulary problem. Moreover, the post-processing can slightly improve the performance of our model.

### The effect of additional features on the development set

We also investigated the effect of three additional features (POS, chunking, and NER features mentioned in "Additional features" section) on the performances of our baseline. In the experiments, the concatenation of basic features and additional features as input is fed into the model, and Table 6 shows the results of different combinations of these features on our development sets.

**Table 6:** The effect of additional features on our development sets

Model	CEMP Dev				GPRO Dev			
	Precision	Recall	F-score	△	Precision	Recall	F-score	△
Baseline	87.58	92.50	*89.97*	–	67.97	86.06	75.95	–
+ POS feature	88.12	91.70	89.87	− 0.10	68.72	85.46	76.18	+0.23
+ Chunking feature	87.21	92.58	89.81	− 0.16	67.21	87.45	76.01	+0.06
+ NER feature	87.57	91.81	89.64	− 0.33	69.32	84.72	76.25	+0.30
+ All features	87.97	91.39	89.65	− 0.32	70.84	83.76	*76.76*	+0.81

Italic values denote the highest values

When the additional features are added, the models achieve slightly lower F-scores than the baseline on the CEMP corpus. The plausible reason is that the deep neural network itself has learned sufficient higher and abstract features automatically from the word and character embeddings with the large training set. However, noise may be introduced into the models by the errors of the NLP tools, which leads to the decrease in performances of the models. On the GPRO corpus, when only the POS feature is added, higher F-score (an improvement of 0.23% in F-score over the baseline) is achieved. The main reason is that the information of POS can help boost the precision of baseline. For example, most entities are nouns but not verbs. When only the chunk feature is added, the model achieves a slight improvement (an improvement of 0.06% in F-score). The main reason is that some entity boundary errors can be revised by the chunking information though some chunking information generated by the GENIA tagger tool is error. The introduction of NER feature alone also improves the performance (an improvement of 0.30% in F-score), which demonstrates that the information of prior entities provided by the GENIA tagger can help boost the performance. When all the additional features are added into the baseline, the best performance (an improvement of 0.81% in F-score) is achieved. Compared with the GPRO training set, the CEMP training set contains more entity mentions (99,623 chemical mentions vs 17,751 gene/protein mentions). The additional features are more helpful for a small training set than large one. For the GPRO task, the different kinds of additional features contribute complementary information, and the introduction of them into our baseline can further improve the performance.

### Performance comparison with other participants on the test set

To further demonstrate the effectiveness of our approach, it is compared with the performance of other CEMP and GPRO track participants. The official CEMP and GPRO top five evaluation results (the best runs per team only) on the test sets are shown in Table 7, where SD denotes the standard deviation of the F-score of each team and teams were grouped based on statistically significant difference between results [30, 32]. The results of team 121 are the results of our BiLSTM-CRF models with the basic feature and the all features on the CEMP and GPRO test sets, respectively (i.e., the best models in Table 6). The results show that our system achieves the highest performances in all teams in the BioCreative V.5 CEMP and GPRO tracks (the F-scores of 90.42% and 79.19%, respectively).

**Table 7:** Performance comparison with other participants on the test sets (the best runs per team)

Row	CEMP Test					GPRO Test				
	Team	Precision	Recall	F-score	SD (%)	Team	Precision	Recall	F-score	SD (%)
A	121(ours)	88.32	92.62	*90.42*	0.25	121(ours)	76.65	81.91	*79.19*	0.10
B	112	88.97	91.82	90.37	0.27	112	75.23	77.49	76.34	0.08
C	107	90.02	90.62	90.32	0.27	153	72.06	80.68	76.13	0.10
D	153	88.02	90.28	89.14	0.30	133	66.53	82.68	73.73	0.10
E	116	84.39	92.97	88.47	0.23	142	74.79	71.63	73.18	0.15

Italic values denote the highest values

### Error analysis

Compared with the results on the CEMP corpus, our model performs poorly on the GPRO corpus. Therefore, we manually analyzed the errors generated by our best model on the GPRO development set. The major errors can be divided into three categories: (1) incorrect boundary, (2) missing gene/protein mention, (3) not a gene/protein mention. An example for each type of error is shown in Table 8.

**Table 8:** Examples of gene/protein named entity recognition errors

Error type	Example
Incorrect boundary	And in the treatment of diseases and conditions that are mediated by *AXL* receptor tyrosine kinase
Missing gene/protein mention	Combination of C1-INH and lung surfactant for the treatment of respiratory disorders
Not a gene/protein mention	Application of tumor inhibitor *MLN4924* to preparation of antiviral drug

The correct entity mentions are underlined, while the misrecognized entity mentions are italicized

For the incorrect boundary error, most cases occur where a gene/protein is nested within a larger gene/protein mention (e.g., our model predicts “AXL” as a mention but the correct mention should be “AXL receptor tyrosine kinase” in Table 8). The main reason may be that the annotated training set contains the tagging inconsistency. For example, “5-ht2a” of the string “5-ht2a serotonin receptor” is annotated as an entity in the document with ID: CN101871931A while “5-ht2a serotonin receptor” is annotated as the same entity in the document with ID: WO2006060762A3. For the missing gene/protein mention error, the reason is that our model cannot detect the entity without sufficient context information. In the example of Table 8, “C1-INH” is the abbreviation of “C1 esterase inhibitor” in the document, but it is difficult to detect the entity in the sentence without sufficient information by our model. In addition, we observed that many strings having similar expressions and strong gene/protein indicators are falsely identified as gene/protein mentions. For example, “MLN4924” consists of uppercase and number, and its context contains the strong gene/protein indicator “inhibitor”. Our model incorrectly identified the chemical as a gene/protein mention. It can be seen from the above analysis, even though automatic learning of high-level features is advantage of deep learning methods and BiLSTM-CRF model can capture long-range dependencies, it is difficult for our model to automatically learn domain knowledge from the raw text and capture sufficient context information from a sentence. Therefore, more contextual information from a document and external knowledge can be considered to improve our model.

## Conclusion

In this paper, we present our system based on a deep learning approach for the chemical and gene/protein NER tasks in the BioCreative V.5 CEMP and GPRO tracks. In our approach, a BiLSTM-CRF model is employed to recognize biomedical entities from patents. Moreover, the effect of additional features (such as POS, chunking, and NER features) for the neural network model is investigated. The experimental results show that the additional features are effective to improve the performance of our system for the GPRO track. And our system achieves the state-of-the-art performances on both CEMP and GPRO corpora. It demonstrates the effectiveness of our approach for biomedical NER task in patents. However, from our error analysis, our system should can be further improved by considering more contextual information at document-level (not only at sentence-level) and external knowledge which will be explored in our future work.
